# Drought decreases incorporation of recent plant photosynthate into soil food webs regardless of their trophic complexity

**DOI:** 10.1111/gcb.14754

**Published:** 2019-08-10

**Authors:** Mathilde Chomel, Jocelyn M. Lavallee, Nil Alvarez‐Segura, Francisco de Castro, Jennifer M. Rhymes, Tancredi Caruso, Franciska T. de Vries, Elizabeth M. Baggs, Mark C. Emmerson, Richard D. Bardgett, David Johnson

**Affiliations:** ^1^ School of Earth and Environmental Sciences The University of Manchester Manchester UK; ^2^ Department of Soil and Crop Sciences Colorado State University Fort Collins CO USA; ^3^ Marine and Continental Waters Program IRTA Sant Carles de la Ràpita Catalonia Spain; ^4^ AgriFood & Biosciences Institute Belfast UK; ^5^ School of Geography, Earth and Environmental Sciences University of Plymouth Plymouth UK; ^6^ School of Biological Sciences and Institute for Global Food Security Queen's University of Belfast Belfast UK; ^7^ Institute of Biodiversity and Ecosystem Dynamics University of Amsterdam Amsterdam the Netherlands; ^8^ Global Academy of Agriculture and Food Security, Royal (Dick) School of Veterinary Studies University of Edinburgh Midlothian UK

**Keywords:** Acari, Collembola, microorganisms, plant–soil interaction, pulse‐labelling, root‐derived C, soil biodiversity, stable isotope

## Abstract

Theory suggests that more complex food webs promote stability and can buffer the effects of perturbations, such as drought, on soil organisms and ecosystem functions. Here, we tested experimentally how soil food web trophic complexity modulates the response to drought of soil functions related to carbon cycling and the capture and transfer below‐ground of recent photosynthate by plants. We constructed experimental systems comprising soil communities with one, two or three trophic levels (microorganisms, detritivores and predators) and subjected them to drought. We investigated how food web trophic complexity in interaction with drought influenced litter decomposition, soil CO_2_ efflux, mycorrhizal colonization, fungal production, microbial communities and soil fauna biomass. Plants were pulse‐labelled after the drought with ^13^C‐CO_2_ to quantify the capture of recent photosynthate and its transfer below‐ground. Overall, our results show that drought and soil food web trophic complexity do not interact to affect soil functions and microbial community composition, but act independently, with an overall stronger effect of drought. After drought, the net uptake of ^13^C by plants was reduced and its retention in plant biomass was greater, leading to a strong decrease in carbon transfer below‐ground. Although food web trophic complexity influenced the biomass of Collembola and fungal hyphal length, ^13^C enrichment and the net transfer of carbon from plant shoots to microbes and soil CO_2_ efflux were not affected significantly by varying the number of trophic groups. Our results indicate that drought has a strong effect on above‐ground–below‐ground linkages by reducing the flow of recent photosynthate. Our results emphasize the sensitivity of the critical pathway of recent photosynthate transfer from plants to soil organisms to a drought perturbation, and show that these effects may not be mitigated by the trophic complexity of soil communities, at least at the level manipulated in this experiment.

## Graphical abstract

How soil food web complexity modulates the response to drought of soil functions related to C cycling and the capture and transfer below‐ground of recent photosynthate C by plants? We provide an exciting ^13^C pulse‐labelling experiment to answer that question. We used experimental mesocosms with one, two or three trophic groups and subjected them to a drought and then to a ^13^C pulse‐labelling. Overall, our results show that drought and soil food web complexity do not interact to affect the soil functions related to C cycling and microbial community composition, but independently affect carbon cycling, with an overall stronger effect of drought.

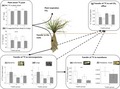

## 1 INTRODUCTION

Drought events are a recurring phenomenon in many ecosystems and are predicted to increase in frequency and intensity in the coming decades (IPCC, 2017; Reichstein et al., 2013). Drought has the potential to severely change ecosystem functioning by impacting plant and soil faunal communities. For example, drought has been shown to alter the composition of grassland plant communities (Cleland et al., 2013; Kardol, Cregger, Campany, & Classen, 2010; Liu et al., 2018) and decrease primary productivity (Reichstein et al., 2013; Schwalm et al., 2010). Furthermore, drought can have considerable effects on the composition and activity of soil microbial communities (de Vries et al., 2018; de Vries & Shade, 2013; Hawkes et al., 2011; Meisner, Deyn, Boer, & van der Putten, 2013), and structure, diversity and activity of soil arthropods (Bardgett & Wardle, 2010; Brose et al., 2012; Kardol, Reynolds, Norby, & Classen, 2011; Santonja et al., 2017; Siebert et al., 2019). These effects on soil organisms generally lead to reductions in ecosystem processes, including litter decomposition and nutrient mineralization (de Vries et al., 2013; Fuchslueger, Bahn, Fritz, Hasibeder, & Richter, 2014; Hagedorn et al., 2016; Santonja et al., 2017; Siebert et al., 2019). However, the effect of drought on the entire soil food web is less clear and contrasting responses of different groups of organisms are sometimes reported (Siebert et al., 2019). Furthermore, drought can modify species interactions within the soil food web. For example, warmer and drier conditions can affect decomposition mediated by trophic interactions (Lang, Rall, Scheu, & Brose, 2014; Thakur et al., 2018) or alter predator‐induced indirect effects on lower trophic levels (Lensing & Wise, 2006), implying that ecological effects of drought will depend on the trophic structure of the food web.

In most terrestrial ecosystems, 80%–90% of photosynthetically fixed carbon (C) ultimately enters the decomposer food web (Gessner et al., 2010). This happens via two main pathways: the decomposition of dead material (above‐ground or below‐ground) or via the transfer of recent photosynthates to the rhizosphere and to mycorrhizal fungi (Bais, Weir, Perry, Gilroy, & Vivanco, 2006; Bardgett, Bowman, Kaufmann, & Schmidt, 2005). There is increasing evidence that C from recent photosynthate allocated to roots and associated mycorrhizal mycelium is of major importance for soil food webs (de Vries & Caruso, 2016; Eissfeller et al., 2013; Goncharov, Tsurikov, Potapov, & Tiunov, 2016; Kanters, Anderson, & Johnson, 2015; Pollierer, Langel, Koerner, Maraun, & Scheu, 2007; Ruf, Kuzyakov, & Lopatovskaya, 2006). For example, mycorrhizal fungi act as a rapid conduit for energy and nutrient supply between the plant and fungal feeding Collembola, such as *Protaphorura armata* Tullberg (Endlweber, Ruess, & Scheu, 2009; Johnson et al., 2005). However, perturbations such as drought can severely alter below‐ground C allocation, which can in turn affect microbial activity and C turnover (Fuchslueger et al., 2014; Hagedorn et al., 2016; Hasibeder, Fuchslueger, Richter, & Bahn, 2015; Ruehr et al., 2009).

Soil communities form complex networks of direct (e.g. feeding interactions) or indirect (e.g. trophic cascades) interactions, and these networks are increasingly recognized as driving key processes in soils (Bardgett & Wardle, 2010). For example, the biomass and activity of fungi, which are the primary drivers of C and mineral nutrient cycling, are greatly influenced by grazing activities of Collembola or Acari (Bardgett & Wardle, 2010; Mikola, Bardgett, & Hedlund, 2002; Nielsen, Ayres, Wall, & Bardgett, 2011). As a consequence, these grazing activities can alter rates of decomposition, soil respiration and carbon and nitrogen mineralization (Bardgett & Wardle, 2010; Mikola et al., 2002; Scheu & Setälä, 2002). These interactions can also cascade through food webs, for example, via predation of detritivores, predators can in turn affect microbial activity by controlling the detritivore abundance and grazing activity (Bardgett & Wardle, 2010; Mikola et al., 2002). The diversity of soil organisms plays a key role in simultaneously mediating many ecosystem processes and driving ecosystem multifunctionality (Wagg, Bender, Widmer, & Heijden, 2014), which raises the possibility that different food web components maximize different ecosystem processes in space and time (Gamfeldt, Hillebrand, & Jonsson, 2008; Hector & Bagchi, 2007). For example, the diversity within and across trophic groups influences the mineralization and sequestration of C and energy pathways (Gessner et al., 2010). Soil biodiversity is under growing threat from a range of disturbances associated with global change (Handa et al., 2014; Wagg et al., 2014), which is of concern since biodiversity loss is a major driver of changes in ecosystem function (Bardgett & van der Putten, 2014). Recent empirical and theoretical evidence has shown that soil biodiversity, and the structural asymmetry of C fluxes through food web, can modulate the resistance and resilience of terrestrial ecosystems to perturbations (de Vries et al., 2012,2018; Rooney & McCann, 2012; Rooney, McCann, Gellner, & Moore, 2006; Schwarzmüller, Eisenhauer, & Brose, 2015; Yang, Wagg, Veresoglou, Hempel, & Rillig, 2018). The difficulty of manipulative reconstructions of soil food webs make experimental studies with soil fauna scarce, and often the effects on soil functions are not consistent (Cole, Dromph, Boaglio, & Bardgett, 2004; Cortet, Joffre, Elmholt, & Krogh, 2003; Lang et al., 2014; Liiri, Setala, Haimi, Pennanen, & Fritze, 2002). Moreover, the scarcity of manipulative studies means that we have poor understanding of how soil food webs impact critical processes related to C cycling, or whether they can buffer the effects of perturbations on soil functions.

Here, our goal was to test how soil food web trophic complexity impacts microbial community structure, soil functions related to C cycling and the capture and transfer below‐ground of recent photosynthate C by plants and whether soil food web trophic complexity modulates the response of these functions to drought. We used microcosms containing soil from semi‐natural acid grassland and examined how reconstructed food webs with one, two or three trophic groups, with or without drought, influence microbial communities, mycorrhizal fungal abundance, decomposition and soil CO_2_ efflux. In addition, we quantified the flux of C from a common temperate grassland plant species, *Agrostis capillaris*, to soil organisms using ^13^C stable isotope labelling. We hypothesized that: (a) greater trophic complexity of soil food webs will increase litter decomposition due to the complementarity of organisms, leading to a higher nutrient availability in soil, and hence an increase of plant growth and C allocation below‐ground; (b) drought decreases plant photosynthesis and C allocation below‐ground, reducing the availability of recently fixed plant C for soil organisms; and (c) soil food webs with fewer trophic groups will be less resistant to drought, leading to a stronger effect of the drought on soil functioning and soil fauna biomass.

## 2 MATERIALS AND METHODS

### 2.1 Construction of the microcosms

Microcosms were constructed using soil collected from a semi‐natural temperate grassland with a history of occasional grazing but no fertilizer application, at the Glensaugh Research Farm, Aberdeenshire, Scotland (56°53′38″N 2°32′29″W). The soil is a humus‐iron podsol derived from Old Red Sandstone and acid igneous rocks (pH ~ 5.47; 11.4 C%; 0.76 N%), and the vegetation is classified as *Agrostis*‐*Festuca* grassland, which is widespread in upland regions of the United Kingdom (UK National Vegetation Classification U4a; Rodwell, 1998). Before potting, soil was defaunated by two consecutive freezing events at −80°C for 24 hr (Johnson et al., 2005), sieved to 4 mm, homogenized and air‐dried. Microcosms were constructed from PVC pots (9 cm internal diameter × 6 cm depth). The base was filled with clay balls to improve drainage, covered with 100 µm mesh to retain animals and 200 g of dry soil was added. Seeds of *A. capillaris* (Emorsgate Seeds), the dominant grass species of these grasslands, were planted in trays filled with a mixture of defaunated soil and chopped fresh roots of mixed species from the same field site to promote colonization of roots by arbuscular mycorrhizal (AM) fungi. After a 20 day initialization period, nine seedlings of *A. capillaris* were transplanted into each pot. Each pot was inoculated with 10 ml of filtered (10 µm) soil slurry (1:2 soil water mix) to promote establishment of native soil microorganisms (Cole, Dromph, et al., 2004). The microcosms were incubated in a controlled environment cabinet (Weiss Technik UK LiAcarid) at 18°C with a light/dark cycle 18/6 hr, and soil moisture was maintained at 60% (w/w) throughout the experiment. Microcosms were preincubated for 14 days under these conditions to allow microorganisms to colonize the soil, and the plants to extend their root systems. After that time, 1 g of grass litter (with a concentration of C of 47 ± 0.3% and N of 4.8 ± 0.2%) was placed on top of the soil in each microcosm providing both a habitat and resource to organisms. Senescent grass leaves, composed mainly of *A. capillaris*, were collected from the same site as the soil, homogenized and air‐dried for 5 days. For the decomposer and the predator trophic groups, we used one species of Collembola, *Protaphorura armata* (Onychiuridae) and one species of gamasid Acari, *Stratiolaelaps scimitus* (formerly *Hypoaspis miles*) respectively. The euedaphic collembolan *P. armata* is an eyeless and nonpigmented species that reproduces parthenogenetically, and is common in UK grasslands (Cole, Buckland, & Bardgett, 2005; Hopkin, 2007). The species is frequently used as an ecologically relevant model organism to study invertebrate–fungus grazing interactions (Cole, Staddon, Sleep, & Bardgett, 2004; Johnson et al., 2005; Scheu & Simmerling, 2004). The gamasid *S. scimitus* is a widespread and common soil mite and is known to feed on a range of different soil‐dwelling prey species including Collembola. Collembolans were extracted from soil collected at the Glensaugh Research Farm; the soil was spread on a tray and individuals of *P. armata* where gathered with pooters and transferred to polypropylene pots with a mixture of plaster of Paris and charcoal (9:1) in the base. Collembolans were kept at 18°C and fed with yeast prior to the experiment. The predatory Acari, *S. scmitidus*, was supplied by Koppert UK Ltd. We constructed three food web treatments: only microorganisms; microorganisms + Collembola; microorganisms + Collembola + predatory Acari. We placed 100 individuals of *P. armata* (equivalent to 15,625 ind/m^2^) and 20 individuals of *S. scmitidus* (equivalent to 3,125 ind/m^2^) in the corresponding treatment pots, which is consistent with natural densities and ratios of predator:prey at Glensaugh (16,092 Collembolans/m^2^ and 7,985 gamasids/m^2^). The organisms were added to the microcosms and a transparent plastic sheet (20 cm high) was wrapped around the rim of each pot to prevent animals escaping.

The experiment comprised a 3 (trophic groups) × 2 (drought vs. control) factorial design with six replicates per treatment. Six supplementary microcosms with the full food web treatment were constructed and were used to determine ^13^C natural abundance in major pools and fluxes. All microcosms (36 microcosms + 6 for ^13^C natural abundance) were randomly interspersed in a controlled environment growth chamber, and locations were swapped randomly and regularly. Each microcosm was wetted with distilled water every 2 days to maintain their original weight. After 68 days of incubation, 7 days of drought was applied to half of the replicates to reach a 25 ± 2.04% moisture reduction based on average moisture of control microcosms, which reflects the effects of chronic summer drought on UK upland grasslands (e.g. Grime et al., 2008). *A. capillaris* was clipped to a height of 10 cm at 29 and 49 days after their transplantation; the biomass clipped was dried, weighed and summed to obtain the final plant biomass.

### 2.2 Isotopic labelling

At the end of the drought, all droughted pots were watered lightly with 20 ml to release the drought and encourage photosynthetic activity; control pots were also watered with 20 ml for consistency. Immediately following, microcosms were randomly assorted into two plastic wooden‐framed chambers (60 × 60 × 50 cm) which received a continuous flow of air containing 99 atom% ^13^C‐CO_2_ at 400 cm^3^/m^3^ for 7 hr into the chambers. Immediately after labelling, and each day for three consecutive days, approximately 20 mg of plant shoots (<2% of total shoot biomass) was harvested from each microcosm. To capture the release of ^13^C‐CO_2_ from soil CO_2_ efflux, an Eppendorf tube containing 0.5 ml of 2 M NaOH was placed on the soil surface inside a small tube (2 cm diameter × 4 cm height) inserted 1 cm into the soil, and the tube was closed for 24 hr and replace periodically for 3 days following the ^13^C labelling. A 0.1 ml aliquot of NaOH was transferred to hydrogen‐flushed exetainers (Labco, UK) and 0.5 ml of 1.3 M H_3_PO_4_ was used to acidify the solution 1 day before the analysis of the ^13^C/^12^C ratio of the accumulated CO_2_ on a 20/20 isotope ratio mass spectrometer (Sercon Ltd coupled to an ANCA TGII gas preparation module). After 3 days, microcosms were harvested destructively. Plant shoots were collected and the experimental litter that was applied to the surface was picked with tweezers and freeze‐dried for analysis of nutrient concentrations and mass loss. Litter mass loss was calculated as the difference of the initial and final litter dry weight. A subsample of soil was taken for the analysis of hyphal length, mycorrhizal colonization of roots and phospholipid fatty acid (PLFA) concentrations. From the remaining soil, animals were extracted by Berlese funnel over 5 days. They were counted under a dissecting microscope, dried and transferred into tin cups for weighing and ^13^C analysis. The six supplementary microcosms that were not ^13^C‐labelled were used to determine the ^13^C natural abundance signature of each C pool. Shoot material and organisms were dried at 60°C for 48 hr and weighed. All samples for C analysis were ground and analysed for total C content and δ^13^C using an elemental analyser (PDZ Europa ANCA‐GSL, Sercon Ltd) coupled to a 20–20 isotope ratio mass spectrometer (Sercon Ltd).

### 2.3 Mycorrhizal colonization and hyphal length

Analysis of *A. capillaris* root colonization by AM fungi was determined by calculating the percentage root length colonization (%RLC). After washing, roots were cleared by boiling for 5 min in a 10% KOH solution, rinsed and stained for 3 min in a boiling ink–vinegar solution (5% ink) (Vierheilig, Coughlan, Wyss, & Piche, 1998). Stained roots were then mounted on slides and fixed with 50% glycerol. Observations were done under a microscope at ×200 magnification and per cent root colonization by arbuscules, vesicles and total AM fungal colonization (including hyphae, arbuscules and vesicles) were quantified using the magnified intersect method (McGonigle, Miller, Evans, Fairchild, & Swan, 1990).

Total length of fungal hyphae was measured in soil extracts using the membrane filter technique modified after Hanssen, Thingstad, Goksøyr, and Goksoyr (1974), followed by staining the hyphae with calcofluor white M2R fluorescent brightener (Sigma‐Aldrich) (Bloem, Bolhuis, Veninga, & Wieringa, 1995) and quantification with the grid‐line intersect method (Brundett, Bougher, Dell, Grove, & Malajczuk, 1996). Briefly, 10 g (fresh weight) of soil in 95 ml of dH_2_O was blended, 5 ml of the soil slurry was diluted with 5 ml of dH_2_O and then 1 ml of the diluted soil slurry was added to 1 ml of formalin, 1 ml of Calcofluor and 7 ml of dH_2_O. Samples were incubated in the dark at room temperature for 2 hr and stored at 4°C until further processed. Two millilitres of these samples was filtered through a 25 mm diameter black 1 µm pore size polycarbonate filter (Osmonics Inc.) and rinsed three times with 3 ml of dH_2_O. The filters were placed on slides with immersion oil and observed with a gridded ocular lens for a total of 50 fields at ×100 magnification under a UV illuminated microscope (Olympus BX61). Hyphal length (*H*) was calculated using the equation *H* = (*I*π*A*)/(2*L*), where *I* is the average number of intersections per grid, *A* is the grid area and *L* is the total length of the grid lines. Then, the total length of fungal hyphae (*F*) (m/g soil) was calculated using the equation *F* = *H*10^−6^ (*A*/*B*) (1/*S*) where *A* is the area of the filter, *B* is the grid area and *S* is the amount of soil filtered (Bloem et al., 1995).

### 2.4 Soil microbial community analysis

The characterization of soil microbial communities was estimated using PLFA analysis, which were extracted using 19:0 phosphatidylcholine (Avanti Polar Lipids) as an internal standard added at the beginning of the extraction procedure for quantitative analysis (Buyer & Sasser, 2012). Fatty acids were extracted from 0.5 g of dry soil in Bligh‐Dyer extractant, containing the internal 19:0 standard, for 2 hr rotating end‐over‐end. The liquid phase was then collected after centrifugation, 1.0 ml chloroform:water (1:1) was added and the lower phase siphoned off and used for lipid separation. Lipids were separated by solid‐phase extraction (SPE) using a 96‐well SPE plate (100 mg silica, Phenomenex) with chloroform, acetone and 5:5:1 methanol:chloroform:water. Fatty acids were then transesterified and extracted. The δ^13^C values of individual PLFAs and their quantification (Thornton, Zhang, Mayes, Högberg, & Midwood, 2011) were analysed by GC–C–IRMS using a Trace GC Ultra gas chromatograph with combustion column attached via a GC Combustion III to a Delta V Advantage IRMS (Thermo Finnigan). In summary, 36 PLFAs were identified in these samples, of which 20 microbial‐specific PLFAs comprising approximately 80% of the total concentration were used in subsequent data analysis. The fatty acids i15:0, a15:0, i16:0 and i17:0 were used as biomarkers for Gram‐positive bacteria; 16:1ω7, 18:1ω7, cy17:0 and cy19:0 were used as biomarkers of Gram‐negative bacteria; and 15:0 and 17:0 were used as general bacterial markers (Frostegård, Bååth, & Tunlio, 1993). The fatty acids 10Me17:0 and 10Me18:0 were used as specific biomarkers of actinomycetes, and 17:1ω8c and 19:1ω8 were used as biomarkers of methane‐oxidizing bacteria (Frostegård et al., 1993). Gram‐positive, Gram‐negative and general bacterial markers were summed to give total bacterial PLFA, and 18:2ω6,9 was used as a marker of fungi (Bååth, 2003; Bååth & Anderson, 2003). The δ^13^C value of each PLFA molecule was corrected for the C added during derivatization using the formula:

$$\delta^{13}C_{\text{PLFA}}=\frac{C_{\text{FAME}\;}\times \delta^{13}C_{\text{FAME}}-C_{\text{MeOH}\;}\times \delta^{13}C_{\text{MeOH}}}{C_{\text{PLFA}}},$$

where C_FAME_, C_MeOH_, and C_PLFA_ denote the number of carbon atoms in FAME, methanol and PLFA, respectively, and δ^13^C_FAME_ and δ^13^C_MeOH_ are the measured ratio of stable isotopes ^13^C/^12^C (in part per thousand, ‰) of FAME and methanol respectively (methanol δ^13^C = −29.3‰). While the fungi are represented by only one PLFA, the bacterial community is represented by several PLFAs. To calculate an overall ^13^C enrichment (atom% excess) of bacterial PLFA, the net ^13^C of all individual (a) bacterial PLFA were summed and divided by the sum of the net C of all individual bacterial PLFA using the following equation:

$${\%}^{13}C_{\text{BacterialPLFA}}=\frac{\sum \left(C_{{\text{PLFA}}_{i}}\times \text{Atom}{\%}^{13}C_{{\text{PLFA}}_{i}}\right)}{\sum C_{\text{PLFA}}}.$$

### 2.5 Calculations

PLFAs were converted to biomass C using the following factors: 363.6 nmol of total bacterial PLFA = 1 mg bacterial C (Frostegård & Bååth, 1996), 11.8 nmol of the PLFA 18:2ω6,9 = 1 mg of fungal C (Klamer & Bååth, 2004). All ^13^C data were converted from δ^13^C values (‰) to ^13^C values (atom%) and then in ^13^C atom% excess subtracting the %^13^C of unlabelled controls from each enriched sample (Johnson, Vachon, Britton, & Helliwell, 2011). The net incorporation of the ^13^C tracer into the different compartment for each pot was calculated as:

$${\text{Net}\;}^{13}C_{\text{pool}}=C_{\text{pool}\;}\times \text{Atom}{\%}^{13}C_{\text{pool}},$$

where C_pool_ is the stock of C in each pool (mg per pot) and Atom%^13^C_pool_ is the atom% excess of ^13^C in each pool.

The C budget was calculated using the net ^13^C excess, and allowed investigation of the dynamics of ^13^C allocation within individual C pools. This C budget was expressed as a percentage of the net ^13^C exported from the plant shoots (via either shoot respiration or allocation to other organs) to the different C pools during the 3 days after labelling calculated using the equation:

$${\%}^{13}C_{\text{transferred}}=\left({\text{Net}\;}^{13}C_{\text{pool}}÷{\text{Plant Net}\;}^{13}C_{\text{exported}}\right)\times 100,$$

where C_pool_ is the stock of C in each pool (mg per pot) and Plant net ^13^C_exported_ is the stock of ^13^C exported by the plant.

### 2.6 Statistical analyses

The experimental design included two fixed factors: treatment (drought or control) and food web trophic complexity (three levels). For all measured variables, two‐way analysis of variance (ANOVA) was used to test the effects of the drought treatment and food web trophic complexity, followed by Tukey post hoc pairwise comparisons. Normality and homoscedasticity of the data were first checked using Anderson Darling and Levene tests respectively. If the assumptions were not met, data were log‐transformed to meet the test assumptions before performing statistical tests. Data from the six additional pots with the full food web that were established and used primarily for natural abundance determination were combined with data from the main experiment for analysis of responses not involving analysis of ^13^C. All statistical analyses were performed using the R software (version 3.4.3, R Core Team, 2017). Statistical tests used α = 0.05 to determine statistical significance.

## 3 RESULTS

Both Collembola and Acari were present at the final harvest, showing that consumers and predators established populations. The number of individual Collembola ranged from 8 to 234 (median = 66) and Acari ranged from 3 to 27 (median = 5) per pot. Generally, we found significant main effects of the drought and the number of trophic groups rather than their interaction on the different response variables (Table 1).

**Table 1:** Results of two‐way ANOVAs for the effects of the drought (drought or control) and food web trophic complexity (one, two or three trophic groups) and their interaction on the processes, soil biota and ^13^C carbon budget

Variables	Drought		Trophic groups (TG)		Drought × TG	
	*F*	*p* value	*F*	*p* value	*F*	*p* value
Process						
Litter mass loss	6.74	**.014**	6.07	**.005**	1.55	.226
Soil CO_2_ efflux	9.27	**.004**	3.31	**.048**	0.03	.976
Soil biota						
Bacterial PLFA	3.65	.060	1.84	.180	0.60	.556
Fungal PLFA	0.63	.433	1.66	.203	0.06	.940
f/b ratio	9.22	**.005**	2.05	.144	0.38	.687
% root colonized by AM fungi	1.25	.270	0.23	.793	1.12	.339
% arbuscules	4.91	**.033**	1.21	.311	1.32	.281
Collembola biomass	38.95	**.000**	8.20	**.008**	0.48	.496
Acari biomass	1.72	.210	—	—	—	—
Carbon budget						
Plant shoot ^13^C net (mg)	5.83	**.022**	0.38	.687	0.10	.903
Plant shoot ^13^C exported after 3 days (%)	19.235	**.000**	0.454	.640	0.307	.738
Net ^13^C transfer from plant export to respiration	0.11	.747	0.294	.748	2.177	.132
Net ^13^C transfer from plant export to fungal biomass	0.18	.677	0.745	.484	0.147	.864
Net ^13^C transfer from plant export to bacterial biomass	0.25	.622	0.31	.736	0.241	.787
Net ^13^C transfer from plant export to Collembola	8.12	**.010**	1.93	.180	0.01	.934
Net ^13^C transfer from plant export to Acari	0.19	.674	—	—	—	—

Note

Significant treatment effects (*p* < .05) are in bold.

Abbreviations: AM, arbuscular mycorrhizal; PLFA, phospholipid fatty acid.

### 3.1 Effects of food web trophic complexity and drought on plant productivity, soil communities and soil functions

**Figure 1:**
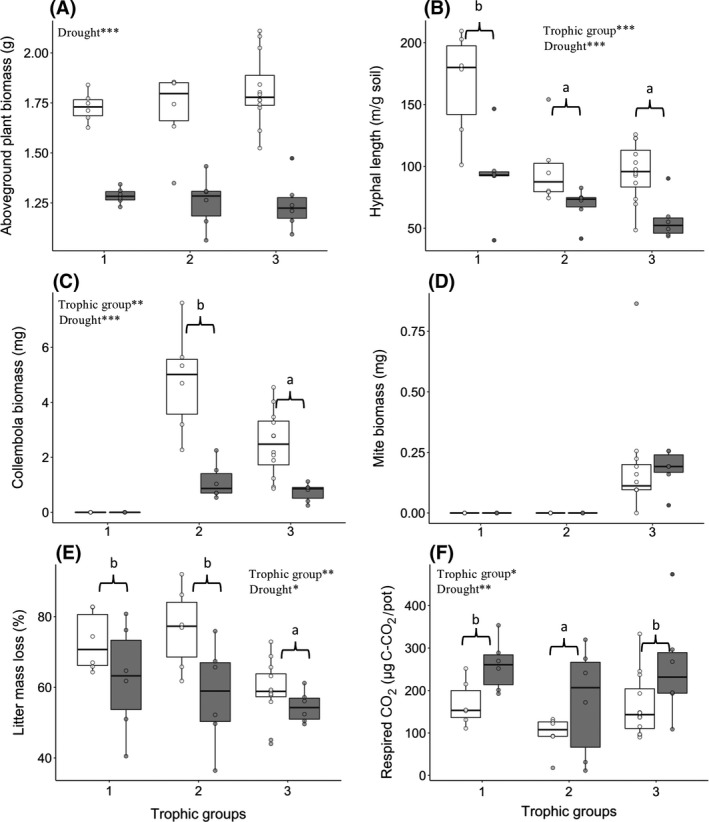
Above‐ground plant biomass (A), total fungal hyphal length (B), Collembola biomass (C), Acari biomass (D), litter mass loss (E) and soil CO_2_ efflux (F) in response to food web trophic complexity (1: microorganisms, 2: microorganisms + Collembola or 3: microorganisms + Collembola + Acari) and drought (control in white and drought in grey). Lines in boxes represent median, top and bottom of boxes represent first and third quartiles, and whiskers represent the largest value no further than 1.5 interquartile range; dots represent single observations. Only the significant terms of the ANOVA are presented (*p < .05; **p < .01; ***p < .001). Tukey comparison between trophic groups are indicated by different letters, a < b

Total hyphal length was reduced by 36% in the presence of Collembola only, and 41% in the presence of Collembola and Acari compared to the microorganisms only (*p* < .001, *F* = 15.09, *df* = 2, *n* = 42, Figure 1b, Table 1). The biomass of Collembola was reduced by 41% in the presence of predators (*p* = .008, *F* = 8.20, *df* = 1, *n* = 30, Figure 1c, Table 1). Above‐ground plant biomass was not influenced by food web trophic complexity (Figure 1a, Table 1), whereas litter mass loss was slower in mesocosms with three trophic groups compared with one or two trophic groups (*p* = .005, *F* = 6.07, *df* = 2, *n* = 42, Figure 1e, Table 1), and soil CO_2_ efflux was less in mesocosms with two trophic groups than with one or three trophic groups (*p* = .048, *F* = 3.31, *df* = 2, *n* = 42, Figure 1c, Table 1). Trophic complexity had no significant impact on fungal or bacterial PLFAs, but there was a trend of decreasing fungal PLFA with two or three trophic groups (Tables 1 and 2). Likewise, the colonization of roots by AM fungi was not influenced by food web trophic complexity (Tables 1 and 2).

**Table 2:** Bacterial and fungal PLFA, fungal/bacterial PLFA ratio and AM fungal root colonization

Variables	Drought	ANOVA	Trophic groups		
			1	2	3
Bacterial PLFA (nmol/g of soil)	Control		1,417 ± 260	1,043 ± 241	1,451 ± 150
	Drought		2,222 ± 499	1,461 ± 277	1647 ± 300
Fungal PLFA (nmol/g of soil)	Control		34.17 ± 10.45	21.55 ± 8	19.37 ± 4.52
	Drought		24.32 ± 6.91	15.13 ± 3.36	15.16 ± 3.04
Fungal/bacterial PLFA ratio	Control (b)	**	0.015 ± 0.002	0.018 ± 0.004	0.013 ± 0.002
	Drought (a)		0.01 ± 0.001	0.01 ± 0.001	0.009 ± 0
% root colonized by AM fungi	Control		59.8 ± 2	65.8 ± 4.88	60.23 ± 2.27
	Drought		57.03 ± 4.74	56.22 ± 3.66	61.39 ± 4.56
% arbuscules	Control (b)	*	28.55 ± 2.88	30.07 ± 3.76	23.2 ± 1.55
	Drought (a)		20.36 ± 3.01	21.58 ± 2.95	22.13 ± 2.76

Note

Values are presented as mean ± *SE*. Significant differences among drought treatment are indicated by asterisks (*p < .05; **p < .01) and the Tukey comparison with different letter with a < b. Results of the ANOVA are in Table 1.

Abbreviations: AM, arbuscular mycorrhizal; PLFA, phospholipid fatty acid.

Overall, drought had a consistent effect on the response variables regardless of soil food web trophic complexity, as reflected by the absence of any significant interaction between the two factors. Drought treatment reduced plant above‐ground biomass by an average of 27 ± 2%, and litter mass loss by 16 ± 5% compared to the controls (Figure 1a,e, Table 1). Soil CO_2_ efflux increased by 63 ± 8% after the release of the drought compared to controls (Figure 1f, Table 1). Drought had no significant impact on the fungal PLFA 18:2ω6,9, but there was a marginal, but not significant increase in bacterial PLFA under drought relative to controls (*F* = 3.65, *df* = 1, *n* = 42, *p* = .064) (Tables 1 and 2); as such, the fungal/bacterial ratio was reduced (*F* = 9.22, *df* = 1, *n* = 42, *p* = .005) by drought (Tables 1 and 2). While the colonization of roots by AM fungi was not impacted by drought, the production of arbuscules was significantly reduced by drought by 21 ± 8% relative to the control (Tables 1 and 2).

### 3.2 Effects of food web trophic complexity and drought on pools and fluxes of ^13^C

**Figure 2:**
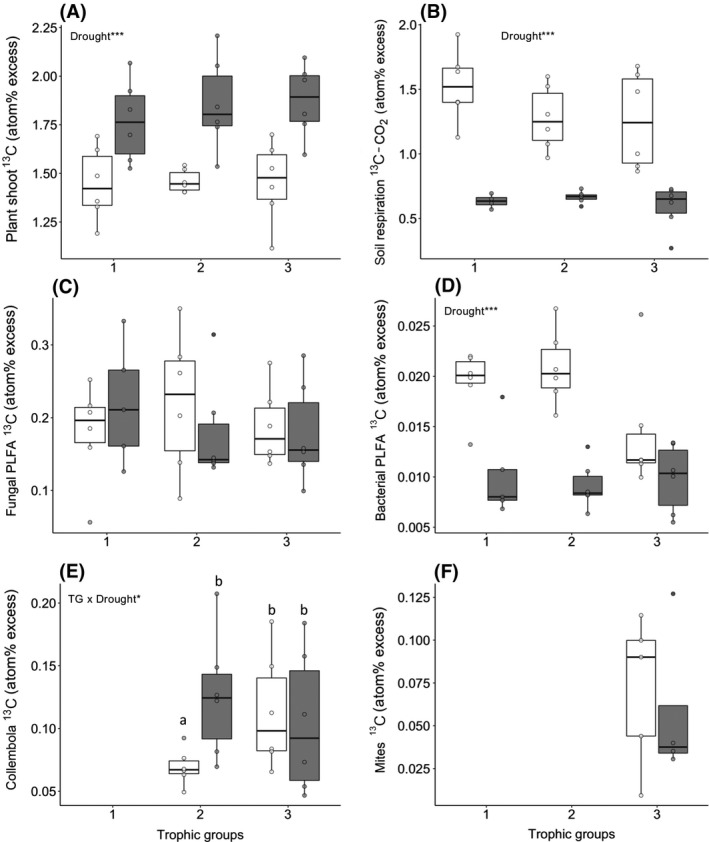
^13^C enrichment of the different carbon pools as a function of the food web trophic complexity (one, two or three trophic groups) and drought (control in white and drought in grey): Above‐ground plant (A), soil CO_2_ efflux (B), bacterial PLFA (C), fungal PLFA (D), Collembola (E) and Acari (F). Lines in boxes represent median, top and bottom of boxes represent first and third quartiles, whiskers represent the largest value no further than 1.5 interquartile range; dots represent single observations. Only the significant terms of the ANOVA are presented (*p < .05; **p < .01; ***p < .001). Tukey comparison between trophic groups are indicated by different letters, a < b

The number of trophic groups had no effect on ^13^C enrichment of the majority of C pools, except the Collembola C pool under control conditions, which had greater ^13^C enrichment in the presence of predators compared to without predators (treatment × trophic groups interaction, *F* = 4.698, *df* = 1, *n* = 36, *p* = .0425, Figure 2e).

At the end of the pulse‐labelling period, ^13^C enrichment of droughted plant shoots was on average 27 ± 4% greater compared to control plants (*F* = 35.2, *df* = 1, *n* = 36, *p* < .001, Figure 2a). During the first 24h after pulse‐labelling, ^13^C enrichment of the CO_2_ released from droughted soil was on average 49 ± 4% lower relative to the control, regardless of the food web trophic complexity (*F* = 6.9, *df* = 1, *n* = 36, *p* < .001, Figure 2b). PLFAs were significantly enriched in ^13^C 3 days after the pulse‐labelling. The fungal PLFA 18:2ω6,9 had the greatest ^13^C enrichment, reaching a maximum of 0.71 atom% ^13^C excess, while maximal enrichment of bacterial PLFA was 0.14 atom% ^13^C excess for the Gram‐negative PLFA 16:1ω7c. While ^13^C enrichment of fungal PLFA was similar between the droughted and control mesocosms (*F* = 0.04, *df* = 1, *n* = 36, *p* = .84, Figure 2c), ^13^C enrichment of bacterial PLFA was reduced in droughted mesocosms, regardless of food web trophic complexity (*F* = 38.7, *df* = 1, *n* = 36, *p* < .001; treatment × trophic groups interaction, *p* = .09; Figure 2d). The incorporation of ^13^C into Collembola was greater in droughted mesocosms relative to controls, but only in the absence of predators (interaction between treatment × trophic groups, *F* = 4.698, *df* = 1, *n* = 36, *p* = .0425, Figure 2e). There was no difference in ^13^C enrichment of Acari in response to the drought treatment (Figure 2f).

### 3.3 Carbon budget and net C incorporation into the food web

**Figure 3:**
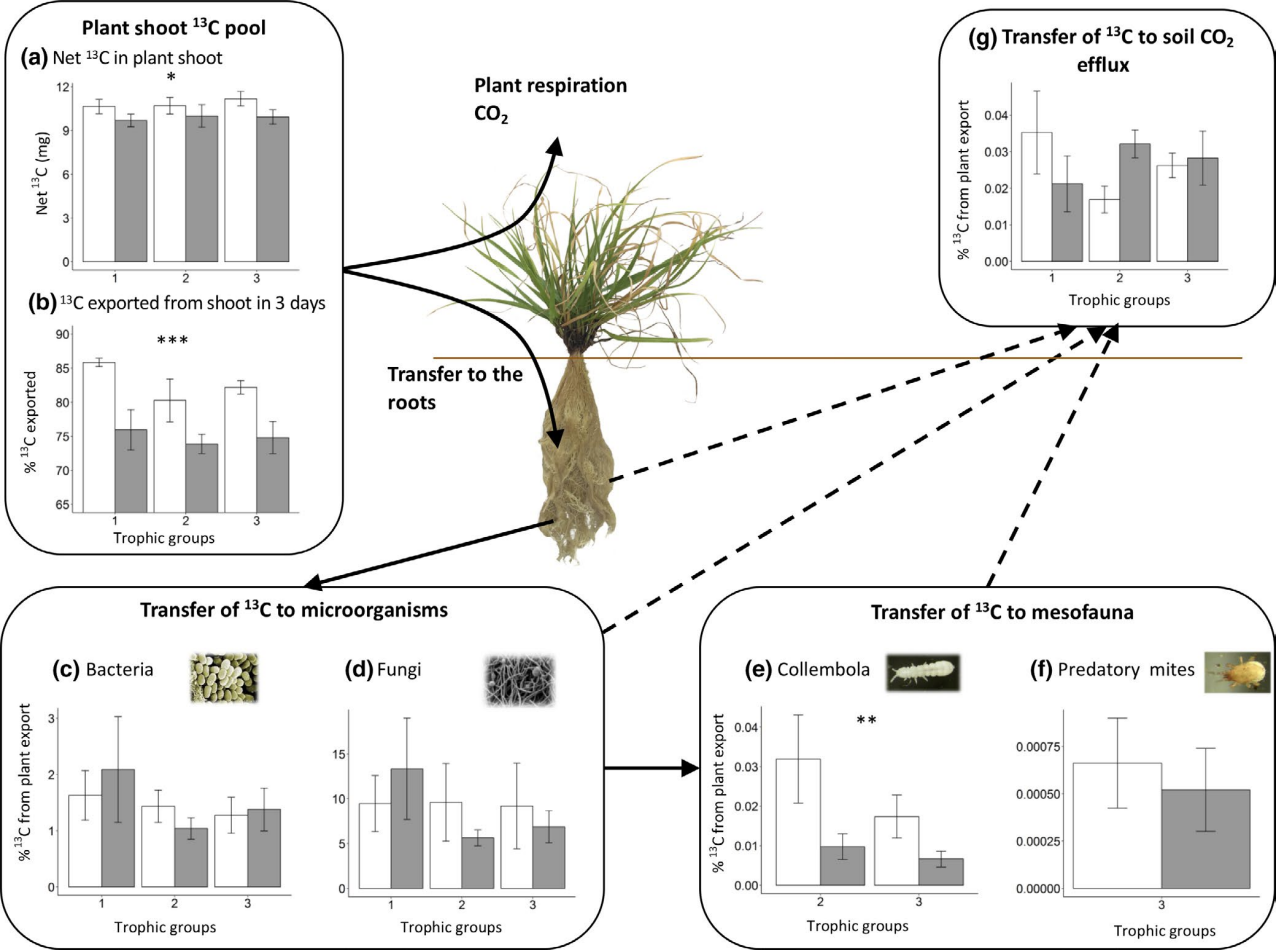
Quantification of the major pools and fluxes of ^13^C labelled recent plant assimilate in response to food web trophic complexity (1, 2 or 3 trophic groups) and drought (control in white and drought in grey). Net ^13^C (mg) in plant shoots immediately after the pulse labelling (a) and ^13^C exported from plant shoots (comprising either allocation to roots or lost as plant respiration) after 3 days expressed as a percentage of the net ^13^C fixed (b). From the amount of ^13^C (mg) exported from plant shoots, we calculate the percentage transfer to different carbon pools comprising bacteria (c), fungi (d), Collembola (e), predatory Acari (f) and soil CO_2_ efflux (g). The soil CO_2_ efflux includes root respiration and was measured during the first 24 hr after the labelling. Mean ± *SE*, significant differences among drought are indicated by asterisks (*p < .05; **p < .01; ***p < .001)

Plants that had been subjected to drought had shoots more enriched in ^13^C immediately after labelling than the controls, but they had overall smaller biomass. Consequently, the net ^13^C in the plant biomass C pool was less in droughted mesocosms than in controls, and was on average 10.8 ± 0.29 mg in the controls and 9.7 ± 0.32 mg in the droughted mesocosms at the end of the labelling period (*F* = 5.829, *df* = 1, *n* = 36, *p* = .02, Figure 3a, Table 1). Three days later, only 1.9 ± 0.32 mg of ^13^C was left in control plant shoots, but 2.5 ± 0.16 mg ^13^C was left in the droughted plant shoots. Therefore, the relative amount of ^13^C exported (through reallocation to the roots or from respiration) from the plant shoots during these 3 days was greater under control compared to drought, with losses of 83 ± 1.19% and 75 ± 1.28% from the initial ^13^C amount respectively (*F* = 19.2, *df* = 1, *n* = 36, *p* < .001, Figure 3b, Table 1). This export of ^13^C was traced into bacteria, fungi, Collembola, Acari and soil CO_2_ efflux. On average across all the treatments, the percentage of the plant ^13^C exported recovered after 3 days in the below‐ground pools was 1.5 ± 0.2% in bacteria, 8.9 ± 1.5% in fungi, 0.02 ± 0.004% in Collembola and 0.0006 ± 0.0002% in Acari (Figure 3c‐f). There were no significant differences in the proportion of recent photosynthate ^13^C transferred to fungi or bacteria in soils with different food web trophic complexities or under drought (Figure 3c,d, Table 1). There was also no significant effect of either drought or food web treatments on the proportion of net ^13^C recovered in Acari or in soil CO_2_ efflux (Figure 3f,g). However, the proportion of recent photosynthate ^13^C exported from plant shoots and recovered in Collembola was consistently lower after drought, decreasing from 0.025 ± 0.006% to 0.008 ± 0.002% respectively (*F* = 8.1, *df* = 1, *n* = 36, *p* = .01, Figure 3e, Table 1). In addition, transfer of ^13^C to Collembola in communities with three trophic levels was almost half of that measured in communities with only two trophic levels (*F* = 1.9, *df* = 1, *n* = 36, *p* = .18, Figure 3e, Table 1). Overall, there was no significant interactive effect of food web trophic complexity on the quantities of ^13^C transferred to the different C pools.

## 4 DISCUSSION

The aim of this study was to test how trophic complexity, manipulated here by increasing the number of trophic levels, moderates the response to drought of soil functions related to C cycling and the capture and transfer below‐ground of recent plant photosynthate C. We found limited evidence for interactive effects between drought and food web trophic complexity; only the transfer of recent photosynthate to Collembola was affected by drought × food web interactions. Therefore, our findings suggest that soil faunal trophic complexity, at least at the level manipulated in this study, does not substantially enhance the resistance of soil functions to drought. Our results show that there was a consistently strong effect of drought on most variables regardless of food web trophic complexity.

We found that drought increased the residence time of recently assimilated C in the leaves, leading to a smaller flux of C from the plant shoots to roots, soil and the atmosphere. Plant‐derived ^13^C was rapidly incorporated into soil microbial communities and mesofauna (Collembolans and predatory Acari), and partially released from the system as soil ^13^C‐CO_2_ efflux. This finding supports the view that numerically abundant and widespread species, such as *P. armata*, are fuelled with recent plant photosynthate via root exudates or mycorrhizal mycelium (Johnson et al., 2005; Pausch et al., 2016; Pollierer, Dyckmans, Scheu, & Haubert, 2012). Incorporation of ^13^C was greater in fungal PLFA than in bacterial PLFAs, with the former representing a larger total C pool when converted into biomass (Frostegård & Bååth, 1996). In our system, this supports the idea that fungi could be key conduits of recent assimilate flow and are critical for transferring energy through higher trophic groups, as suggested by other studies (e.g. Johnson et al., 2005; Pausch et al., 2016).

The food web treatments used in this experiment were effective because Collembola density decreased in the presence of the predatory Acari, and fungal hyphal length, a measure of the production of extramatrical hyphae, decreased in the presence of Collembola, showing a strong cascading top‐down effect of higher trophic levels. In accordance with our first hypothesis, food web trophic complexity regulated litter decomposition and the soil CO_2_ efflux. However, while we expected litter to decompose faster in the presence of more trophic groups, due to complementarity of soil organisms, we observed slower litter decomposition with the three trophic groups compared to only one or two trophic groups. It has been suggested that predation pressure may prevent Collembola from over‐exploiting fungal populations (Hasegawa & Takeda, 1995), which could enhance the rate of litter breakdown. Conversely, predation on Collembola could retard decomposition by preventing the positive effects of Collembola on litter breakdown (Berg & Laskowski, 2005; Cortet et al., 2003). Food web trophic complexity did not influence plant performance, measured in terms of biomass and ^13^C uptake by photosynthesis. This was despite there being significant effects of food web trophic complexity on litter decomposition. There is currently no clear consensus in the literature on plant responses to food web composition. While several correlative studies have shown that the diversity of soil organisms is important to maintain ecosystem functioning, including plant productivity (de Vries et al., 2013; Wagg et al., 2014), other experimental studies have shown little effect of food web composition on plant growth (Bradford et al., 2002; Cole, Dromph, et al., 2004; Liiri et al., 2002). For example, Cole, Dromph, et al. (2004 detected only a small effect of 18 different food web compositions on the growth of *A. capillaris*. The lack of effect of the soil food web trophic complexity on plant growth in our study might be for a number of reasons. For example, it is possible that variation in food web trophic complexity has multiple, but opposing effects on plant growth (e.g. Bradford et al., 2002), and changes in food web trophic complexity might be realized only in the long term.

In contrast to our first hypothesis, ^13^C enrichment and the total amount of C transferred from plant shoots to the other C pools varied little with food web trophic complexity. This finding suggests that variations in the trophic structure at the scale of our study had no influence on the plant C uptake and transfer to microbial communities, despite having some effect on their biomass (as also seen by Cole, Dromph, et al., 2004). However, in the absence of drought, ^13^C‐CO_2_ efflux tended to decrease with the number of trophic groups, suggesting an efficient C transfer through the trophic levels. This transfer of plant‐derived C to higher trophic groups leads to greater retention of C below‐ground as a result of reduced loss via soil respiration.

According to our second hypothesis, our study revealed large effects of drought on several response variables, and most of these responses were likely mediated indirectly through plants and Collembola given the magnitude by which those responded to drought. The physiological and growth responses of plants to drought were complex. For example, despite observing an overall decrease of plant biomass in response to drought, more ^13^C was fixed by plant shoots during the pulse‐labelling immediately after the cessation of the drought. This observation might be explained by either greater ^13^C uptake due to higher photosynthetic activity stimulated by the cessation of drought or by reduced loss of ^13^C from leaf respiration (Atkin & Macherel, 2009) during the pulse‐labelling period. Once fixed by shoots, the ^13^C may be respired, used to produce new shoot material, temporarily stored or allocated below‐ground into roots and to soil organisms (Leake, Ostle, Rangel‐Castro, & Johnson, 2006). Three days after labelling, we observed greater retention of recent photosynthate in plant shoots subjected to drought. These findings are in line with results from beech saplings where drought stress doubled the residence time of fresh assimilates in foliar biomass due to a decrease in phloem transport velocity (Ruehr et al., 2009), and reduced below‐ground C allocation (Fuchslueger et al., 2014; Ruehr et al., 2009).

Under control conditions, soil CO_2_ efflux mainly comprised recent photosynthate, while after drought, a greater proportion of CO_2_ originated from other sources. This result confirms that proportionally less recent photosynthate was invested below‐ground under drought, which might promote a shift of soil microbial community towards increased decomposition of soil organic C (Bradford, Fierer, & Reynolds, 2008), explaining the reduction in ^13^C enrichment of soil CO_2_ efflux. Collectively, our results indicate that the rate of ^13^C transport from leaves below‐ground is slower after a drought perturbation, and thus, the connection between above‐ and below‐ground processes is reduced. Our results confirm that drought increases the time‐lag between photosynthesis and soil CO_2_ efflux (Kuzyakov & Gavrichkova, 2010; Ruehr et al., 2009).

In contrast to theory (Rooney & McCann, 2012; Rooney et al., 2006) and our third hypothesis, manipulation of food web trophic complexity had limited impact on C flow to microorganisms and on buffering the effects of drought on ecosystem processes. However, these theoretical analyses also suggest that asymmetry of pathways of C flow below‐ground, reflected here by recent photosynthate and litter decomposition, is expected to confer stability on ecosystem function. Our findings that drought may weaken the recent photosynthate pathway of C flow below‐ground (i.e. impacting the flux of ^13^C from plant shoots to below‐ground pools and fluxes) suggest it also impacts the asymmetry of C flow through soil food webs. In addition, inclusion of predators reduced the effect of drought on ^13^C transfer to Collembola, indicating the importance of higher trophic groups on C dynamics through food webs. These results provide the basis for further experimental work to distentangle the mechanisms by which drought affects energy flow through, and asymmetry of, different channels.

We observed less ^13^C enrichment of bacterial PLFA after drought, suggesting either that drought leads to bacteria using alternative sources of C to recent assimilate, or that they were less efficient in using this pathway of C. However, this difference was not observed for fungi. Fungi have stronger cell walls preventing water loss, and they can redistribute water by mycelial networks, and so are considered to be more resistant to drought than bacteria (Guhr, Borken, Spohn, & Matzner, 2015). Furthermore, bacteria and fungi often occupy different habitats within soil: bacteria often live within water‐filled pore spaces while most fungi live in air‐filled pore spaces (Ritz 2011). These habitat preferences may help explain why bacteria and fungi were affected by drought differently. In the absence of predators, the ^13^C enrichment of Collembola was less in control conditions compared to drought. This finding could be related to the reduced biomass of Collembola after the drought that likely decreased the competition for ^13^C‐enriched substrates (such as fungal hyphae). By contrast, in the presence of predators, ^13^C enrichment of Collembola did not differ between control and drought treatments. Here, there was overall smaller biomass of Collembola and the difference in biomass between control and drought treatments was less pronounced than in the absence of predators, which could have maintained a lower ratio of Collembola to ^13^C‐labelled substrates thus leading to greater ^13^C enrichment of Collembola. Our results showed no evidence of an impact of drought on the net transfer of C from the plants to microbial communities; however, we observed less net C transfer from plants to Collembola, mainly due to the decrease in biomass reflecting their sensitivity to drought (Hopkin, 1997; Makkonen et al., 2011).

Overall, our study identifies an important effect of drought on plant–soil C fluxes mainly by acting on plant physiology (respiration and C allocation), that regulates the availability of resources to the below‐ground food web, and which may affect food web stability (Rooney et al., 2006). We also showed that an abundant and widespread species of Collembola is highly sensitive to drought, and its feeding activity is moderated by the presence of predators, suggesting the importance of higher trophic groups on C dynamics through below‐ground food webs. The recent photosynthate C pathway is critical to ecosystem functioning and the soil food web, and our work demonstrates its sensitivity to perturbations resulting from global change.

## AUTHOR CONTRIBUTION

MC, DJ and RDB designed the study, with contributions from all the authors and MC and NAS carried out labwork and analysed the data. MC carried out statistical analysis and wrote the manuscript with DJ, and with contributions from all authors. DJ receives partial support from the N8 AgriFood programme. We thank the University of Aberdeen, L. Harrold for isotopic analysis, and the James Hutton Institute, B. Thornton and G. Martin for the PLFA isotopic analysis and access to Glensaugh Farm. We also thank two anonymous reviewers for their helpful suggestions on a previous version of the manuscript.
